# Evaluating the implementation fidelity of basic emergency obstetrics and neonatal care services in Beyeda District, Northwest Ethiopia: a case study evaluation

**DOI:** 10.3389/fgwh.2024.1418338

**Published:** 2024-09-03

**Authors:** Hawltu Abeyu Ejigu, Lake Yazachew, Getasew Amare, Chalie Tadie Tsehay, Asebe Hagos, Tesfahun Zemene Tafere

**Affiliations:** ^1^ Semien Mountains Mobile Medical Service Project, Debark, Ethiopia; ^2^ Department of Health Systems and Policy, Institute of Public Health, College of Medicine and Health Sciences, University of Gondar, Gondar, Ethiopia

**Keywords:** adherence, BEmONC, quality of delivery, participant responsiveness, Ethiopia

## Abstract

**Background:**

Worldwide, nearly 830 women die from complications of pregnancy and childbirth daily. Ninety-nine per cent of these maternal deaths take place in low and middle-income countries. Basic Emergency Obstetric Care (BEmONC) is one method of reducing maternal mortality related to pregnancy and childbirth complications. However, the status of its implementation fidelity has not been investigated. Therefore, this study sought to evaluate the implementation fidelity of BEmONC services in Beyeda District, Northwest Ethiopia.

**Evaluation methods:**

A single case study design with mixed method was employed from June 01 to July 30, 2022. 415 client exit interviews, 14 key informants’ interviews and 423 retrospective document reviews were conducted. Adherence, participant responsiveness and quality of delivery dimensions from Carroll's conceptual framework, with a total of 21 indicators were used in this evaluation. The overall implementation fidelity status of BEmONC service was judged based on the pre-seated judgmental criteria as; low, medium, and high fidelity.

**Results:**

The overall degree of implementation fidelity of the BEmONC services was 74.5%. Moreover, the implementation fidelity status of adherence, quality of delivery, and participant responsiveness dimensions were 74.7%, 77.2%, and 71.5% respectively. Uterotonic drugs were not administered as per the recommended protocol. Participants’ engagement towards the neonatal resuscitation service delivery was inadequate. Likewise, healthcare providers’ respect for the clients was not sufficient. Furthermore, women aged >30 years, being government employed and ANC visits four and above were variables positively associated with the quality of delivery of BEmONC services.

**Conclusion:**

The overall implementation fidelity of the BEmONC services was judged as implemented in medium fidelity. Moreover, the adherence, participant responsiveness and quality of delivery dimensions were found to be implemented in medium fidelity. Therefore, public health sectors at all levels should strive to enhance the implementation fidelity of BEmONC services. Moreover, healthcare providers should adhere to the BEmONC implementation protocol guideline during service delivery. Healthcare managers should facilitate a continuous awareness creation for mothers regarding the merit of neonatal resuscitation for neonatal complications. Furthermore, healthcare providers should give due respect to mothers while delivering the services.

## Background

Every day, almost 830 women worldwide die from pregnancy- and childbirth-related problems (1). Ninety-nine percent of these maternal deaths occur in low- and middle-income nations, and most of them are avoidable (2). Obstetric complications, such as haemorrhage, sepsis, eclampsia, obstructed labor, and fetal distress, remain the leading cause of death for women of reproductive age and newborns in low- and middle-income countries. Treatment and management of obstetric problems in healthcare facilities through the use of Basic emergency obstetric and neonatal care (BEmONC) can avert these deaths (3–6). Sub-Saharan Africa alone accounts for 179,000 pregnancy and childbirth-related deaths each year (1).

Ethiopia has made notable strides in maternal and child survival in the previous 20 years (7). An estimate from the united nations inter-agency group for maternal mortality indicated that Ethiopia's MMR decreased from 953 in 2000 to 267/100,000 in 2020 (8). This improvement is a result of concerted efforts in healthcare policy, infrastructure, and service delivery (9). However, despite these progress, maternal health service metrics remain below the international and national goals (10). In Ethiopia, only half (50%) of childbirths take place at the health institutions (11). As a result, Ethiopia is one of the countries with the highest rates of maternal deaths worldwide (12). The primary causes of maternal mortality in Ethiopia are mentioned to be pregnant-related complications such as haemorrhage obstructed labor, pregnancy-induced hypertension, puerperal sepsis, and unsafe abortions (13). Effective maternal health interventions can avert the majority of causes of maternal health mortality (14).

The primary supply-side factors affecting maternal and neonatal deaths are the lack of hospitals and clinics, the shortage of skilled healthcare providers, the deficient health center referral system, the scarcity of BEmONC medicines and equipment, and the underfunding of the program. Whereas delays in seeking care during pregnancy, childbirth, or for newborn health, low level of education and awareness about maternal and neonatal health, poor women's decision-making power about their health care, financial limits, and cultural and societal norms are some of the obstacles on the demand side (15).

Seven essential obstetric services, often known as “signal functions,” have been determined to be vital to BEmONC: administration of parenteral antibiotics, administration of parenteral uterotonics, administration of parenteral anticonvulsants, manual removal of the placenta, removal of retained products (manual vacuum aspiration), assisted vaginal delivery and resuscitation of the newborn (16). Access to high-quality BEmONC could potentially reduce maternal mortality by up to 75% and neonatal death by up to 40% associated with intrapartum in low-resource settings (17). BEmONC can be provided by skilled birth attendants, such as midwives, and other health care providers trained in basic emergency obstetrics and newborn care at a well-equipped health center (18–21).

Ethiopia continues to face public health challenges due to high maternal and perinatal mortality, despite an increase in BEmONC facilities offered by both government and non-governmental organizations. Many of them die because they were not treated promptly enough in a hospital or because the care they received was insufficient. Some of them perish because they were not admitted until their condition was critical (22–25).

Even though some cross-sectional quantitative studies were discovered about the availability and quality of BEmONC services in Ethiopia (22, 26), there has been much uncertainty regarding the status of the implementation fidelity of these vital maternal health services as a country in general and at Beyeda district in particular. Hence, this study aimed to evaluate the implementation fidelity of BEmONC services in Beyeda district, northwest, Ethiopia.

## Methods and materials

### Evaluation design and settings

An evaluation using a mixed-methods case-study design was carried out from June 1, 2022, to July 30, 2022, in Beyeda District, northwest Ethiopia. A mixed-methods case study design combines both quantitative and qualitative research methods to explore a particular case in depth. The quantitative methods employed in this study were exit interviews and document reviews whereas, the qualitative method included the key informant interviews. This approach of research method allows researchers to gain a comprehensive understanding of the case by using diverse data sources and analytical strategies. Beyeda district was identified as one of the 47 drought-prone and food-insecure districts by the regional government in 1999 because of its inaccessibility and dearth of the most basic infrastructure (27). As a result, utilization of key maternal health services such as Antenatal Care (ANC) visits, delivery in a healthcare facility, and Postnatal Care (PNC) continue to be severely impacted in the district. The total population of the district is 97,559. The district has four public health centers and 22 health posts. The study participants were (a) mothers who received obstetric care services, (b) health care providers working in the maternity units, (C) heads of HCs, and (d) obstetric care services documents.

### Evaluation approach and dimensions

**Figure 1:**
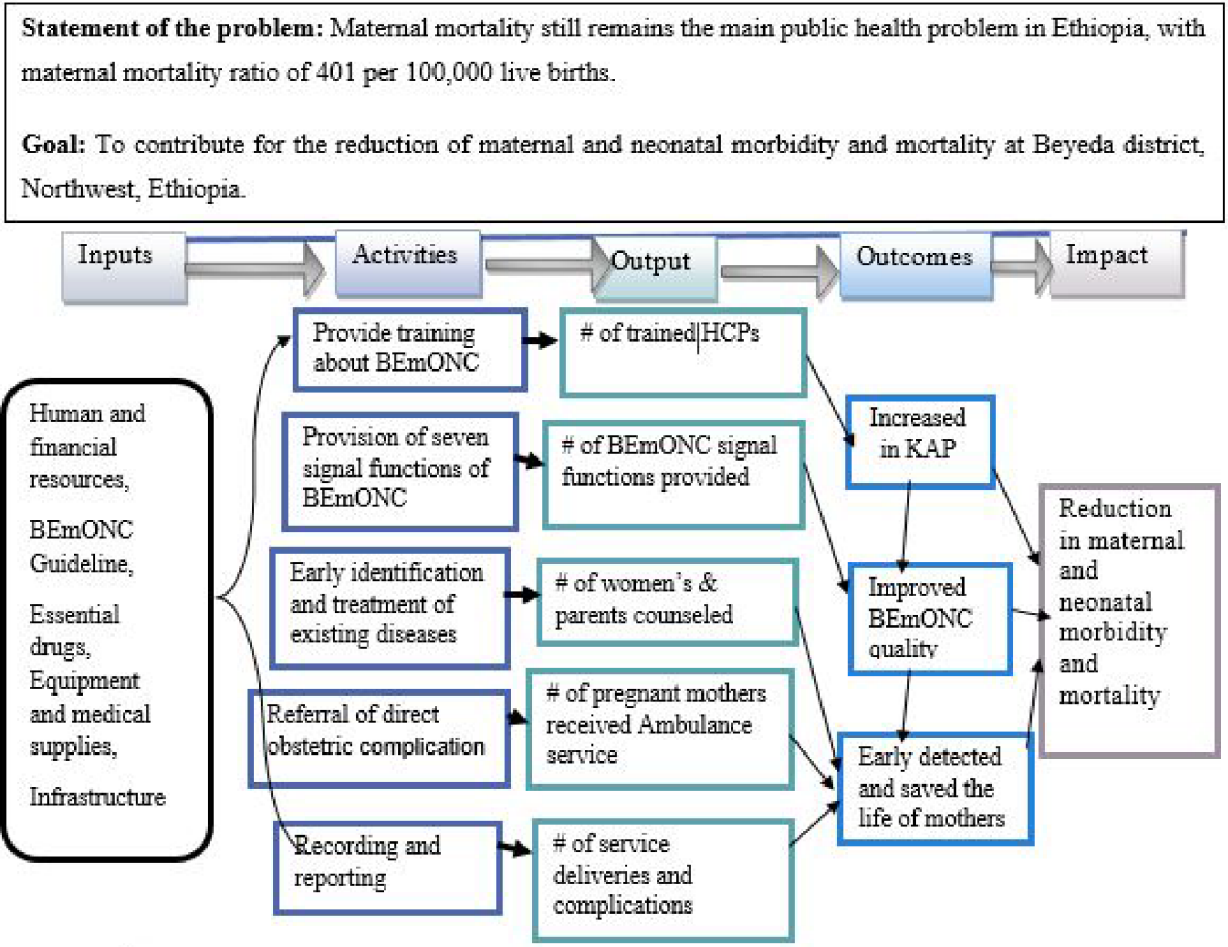
Logic model of BEmONC services.

The implementation fidelity of Basic Emergency Obstetric and Newborn Care (BEmONC) services was assessed using formative evaluation methods. This evaluation used a total of three dimensions, namely adherence, quality of delivery and participant responsiveness which were developed from Carroll's conceptual framework for implementation fidelity (28). Both quantitative and qualitative data were gathered concurrently, analyzed separately, and then combined when the results were interpreted. The program process theory of the implementation fidelity of BEmONC services was the main focus of the evaluation as indicated in the program logic model (Figure 1).

### Variables and measurements

Implementation fidelity is described as the extent to which a given program adheres to the original program model, that is the model that the program developers had intended to use (29). Evaluation dimensions are aspects of program performance to be assessed by the evaluation. Adherence refers to the delivery of program services or interventions as it was designed (30). The adherence dimension was measured using eight indicators to determine the extent to which all the signal functions of BEmONC service were provided to the clients as per the recommended protocol. Participant responsiveness refers to the engagement of the participants in the intervention (30). The participant responsiveness dimension was measured using seven indicators. Quality delivery refers to whether an intervention was carried out in a manner appropriate for accomplishing its goals., i.e., “the extent to which a provider approaches a theoretical ideal while delivering program content” (30). Quality of delivery was assessed using six indicators to measure features of quality of service delivery (31). It was measured with a five-point Likert scale ranging from (1 = strongly disagree to 5 = strongly agree). Then clients’ response was dichotomized based on a threshold score. The demarcation threshold formula was used to calculate the threshold score (32).

$$\left(\frac{\mathrm{Total}\;\mathrm{highest}\;\mathrm{score}-\mathrm{Total}\;\mathrm{lowest}\;\mathrm{score}}{2}\right)+\mathrm{Total}\;\mathrm{lowest}\;\mathrm{score}$$

Accordingly, participants who scored above 60 on the quality of delivery questions were considered as having positive attitudes otherwise negative attitudes towards the service.

The indicators’ weight was calculated using the formula $(\frac{\mathrm{Observed}\;\mathrm{numberX}\;\mathrm{Indicator}\;\mathrm{weight}}{\mathrm{Expected}\;\mathrm{number}}$). The achievement was calculated by $(\frac{\mathrm{Score}}{\mathrm{Weight}}x100$). Then the judgment parameter for the indicators was rated as low, medium, and high fidelity if the score was <60%, 60–84.9%, and >85% respectively.

### Sample size and sampling procedures

The availability of resources was measured using an inventory of four health centers. The sample size for quality of delivery and participant responsiveness was calculated using single population proportion formula: *n* = $\frac{z{\left(\frac{\alpha }{2}\right)}^{2}x\;\;p\;(1-p)}{d^{2}}$ with the assumption of a 5% margin of error (d), 95% confidence level, *p* = 50%, and 10% non-response rate. Then, the final sample size was 415.

All four health centers in Beyeda district were considered for this study to make the finding more representative. Then, the proportional allocation was done to each health center based on the number of the previous two-month client flow in the maternity unit. Finally, a systematic sampling technique was employed and study participants were selected in every 3rd interval.

A six-month documents (patient records/partographs) were reviewed retrospectively to measure the adherence of health care providers to the BEmONC national guideline. Moreover, 14 key informants who were more familiar with the services were purposively selected and interviewed for qualitative data to supplement the quantitative findings.

Women with abortive pregnancy outcomes and delivered mothers within 28 days of postpartum who received at least one of the signal functions of BEmONC services at health facilities of Beyeda District during the data collection period were included in this evaluation. Moreover, the district reproductive health coordinator, health center heads and health center midwifery case team leaders who have worked in the facilities at least for six months also were included. Patient records (documents) which were incomplete were excluded from the study.

### Data collection tools and procedures

This evaluation used a structured questionnaire for the exit interview, checklists for document reviews and semi-structured questions for key informant interviews. The questionnaires were adapted from the Averted Maternal Death and Disability (AMDD) data collection module, WHO BEmONC guidelines and other related literature (33, 34). To maintain consistency and clarity, these tools were originally designed in English, translated into the Amharic native tongue, and then back to English. Two BSC midwife nurses were hired as supervisors and four BSC midwife nurses were hired as data collectors. Both the supervisors and the data collectors received two days of training before engaging in the data collection process. The whole evaluation process was supervised daily and closely. Moreover, a pre-test was conducted on 21 (5%) of participants before the actual data collection and the necessary modification was made. The Cronbach alpha value of 0.76 ensured the reliability of the tools. The qualitative data was collected after debriefing key informants and arranging favourable times, and places for interviews. Each key informant interview was carefully recorded on the field note. Exit interviews were done in the postnatal room to ensure both the auditory and visual privacy of the clients.

### Data management and analysis

Data were cleaned and entered into Epi data version 4.6 and exported to SPSS version 25 software for analysis. A binary logistic regression model was fitted to determine the association between dependent and independent variables. The Hosmer and Lemeshow goodness of fit test was used to assess the model's fitness. In multivariable logistic regression, variables from the bivariable regression with a *p*-value of less than 0.2 were included. Adjusted Odd Ratio (AOR) with a 95% confidence interval (CI) and *p*-value <0.05 were used to declare the significant factors and strength of association. Thematic analysis was used to code and categorize audio-recorded qualitative data after it had been transcribed and converted into text. Then both quantitative and qualitative data were mixed and integrated during the interpretation phase and supplemented the quantitative findings. The weight of each evaluation dimension and the respective indicators which were used to decide the degree of the implementation fidelity of BEmONC were determined by the stakeholders per the relevance for the services (Table 1). Involving stakeholders of the program in determining the weights of healthcare indicators is crucial for ensuring that the final outcomes are representative, balanced, and aligned with the needs and values of different groups. This participatory approach ensures that the weighting process is comprehensive, inclusive, and aligned with the collective values and priorities of the community and healthcare system. Accordingly, the implementation fidelity level of each dimension was judged as low, medium and high if the score was <60%, 60–84.9%, and >85% respectively. The implementation fidelity status of the BEmONC services was then determined by adding the scores for each dimension.

**Table 1:** Stakeholders’ identification and analysis matrix for BEmONC service at Beyeda District, Northwest Ethiopia, 2022.

Stakeholders	Role in the program	Perspective on the evaluation	Role in the evaluation	Means of communication
Amhara region health bureau	Training of health care providers, supplies and supervision, Develop guideline	Service gap identification, Experience sharing and policymaking	Data source	Meeting Discussion Phone
North Gondar zone health department	Conduct review meetings and Supervision	Use of the findings for service improvement	Data source	Key informant interview, Report Telephone
District health office	Budgeting, facilitation of services, community mobilization	Service gap identification	Data source, Facilitating the evaluation process, Technical support	Telephone Meeting
District health facilities	Provider of delivery services	Service gap identification	Data source	Meeting Telephone
Health care providers	Provision of BEmONC service	Use the evaluation findings and recommendations for program improvement	Data source, Technical support	Meeting Telephone Interview
Clients	Recipients of services	Beneficiary from the evaluation findings	Data source	Exit interview

## Results

This evaluation revealed that the overall degree of implementation fidelity of the BEmONC service was 74.5%. Moreover, the implementation fidelity status of adherence, quality of delivery, and participant responsiveness dimensions were 74.7%, 77.2%, and 71.5%, respectively.

### Adherence of health care providers to the BEmONC guideline

The retrospective document review showed that partograph was recorded for 81% of the study participants based on obstetrics management protocol. Similarly, uterotonic drugs, parenteral antibiotics and parenteral anticonvulsants were administered based on the algorism for 58.3%, 81.3% and 69.3% of participants respectively.

Moreover, assisted vaginal delivery, removal of retained products and neonatal resuscitation was performed for 74%, 78.45 and 81.6% of participants respectively based on the protocol. This finding was supported by the qualitative findings.

*“There are now health care providers who have had BEmONC training and those who have not. Providers who have BEmONC training adhere to the guidelines during delivery, the removal of a retained placenta, and the provision of respiratory support to children. For example, what I frequently see on Partograph utilization, is that the partograph was not filed during labour follow-up, rather it would be filled up after the mother gave birth. In this regard, health care providers were not adhering towards the recommended protocol during service delivery” (Health center head in Beyeda district).*

In this evaluation, the overall implementation fidelity of adherence to BEmONC was found to be 74.7% (Table 2).

**Table 2:** Judgment analysis matrix of adherence dimension indicators for evaluating the implementation fidelity of the BEmONC service at Beyeda District, Northwest Ethiopia, 2022.

Indicators	E	O	W	S	A (%)	JP
The proportion of clients whom partograph was recorded based on the protocol	415	336	4	3.24	81.0	High
The proportion of clients who were administered uterotonic drugs based on standards	415	329	6	3.5	58.3	Low
The proportion of clients who were given parenteral antibiotics based on the indication	276	224	4	3.25	81.3	High
The proportion of clients who were administered parenteral anticonvulsants based on the algorism	163	113	4	2.77	69.3	Medium
The proportion of clients for whom removal of the placenta was performed based on standards	183	138	5	3.77	75.4	Medium
The proportion of clients for whom removal of retained products was performed based on standards	168	138	5	4.11	78.4	Medium
The proportion of clients who were assisted during vaginal delivery based on the indication	236	175	5	3.70	74.0	Medium
The proportion of neonates who were resuscitated based on the protocol	212	173	5	4.08	81.6	High
Overall fidelity of adherence			38	28.4	74.7	Medium

E, expected; O, observed; W, weight; S, score [(observed × weight)/expected]; A, achievement in percentage [(S/W) × 100]; JP, judgment parameter.

### Participant responsiveness towards the BEmONC services

This evaluation showed that 76.2%, 76 and 69.3% of the study participants were interested in receiving uterotonic drugs, parenteral antibiotics, and parenteral anticonvulsants respectively. Moreover, 56.4% of study participants expressed an interest in the delivery of neonatal resuscitation. This evaluation revealed that the overall score of the fidelity of participant responsiveness was 71.5% (Table 3).

**Table 3:** Judgment analysis matrix of participant responsiveness dimension indicators for evaluating the implementation fidelity of the BEmONC services at Beyeda District, Northwest Ethiopia, 2022.

Indicators	E	O	W	S	A (%)	JP
Proportion clients who were interested in receiving uterotonic drugs to restore their uterus and prevent bleeding	415	316	6	4.57	76.2	Medium
Proportion clients who were interested in receiving parenteral antibiotics	276	210	4	3.04	76.0	Medium
The proportion of clients who were interested in administering parenteral anticonvulsants	163	113	4	2.77	69.3	Medium
The proportion of clients who were interested in performing manual removal of placenta	183	134	5	3.66	73.2	Medium
The proportion of clients who were interested in performing the removal of retained products	168	128	5	3.81	76.2	Medium
The proportion of clients who were interested in performing assisted vaginal delivery	236	172	4	2.92	73.0	Medium
The proportion of clients who were interested in providing neonatal resuscitation	212	162	5	2.82	56.4	Low
Overall fidelity of participant responsiveness			33	23.6	71.5	Medium

E, expected; O, observed; W, weight; S, score [(observed × weight)/expected]; A, achievement in % percentage [(S/W) × 100]; JP, judgment parameter.

### Quality of delivery of BEmONC services

#### Socio-demographic characteristics of study participants

A total of 415 clients participated in this study. The age of participants in the study ranged from 18 to 45 years and their mean age was 30.04 (SD ±7) years. About 79% of mothers were married, 81.4% were orthodox, 32% were unable to read and write, and 52.5% were housewives (Table 4).

**Table 4:** Socio-demographic characteristics of study participants of the BEmONC services at Beyeda District, Northwest Ethiopia, 2022 (*n* = 415).

Variables	Category	Frequency	Percent
Age in years	<20	33	8.0
	20–24	66	15.9
	25–30	129	31.1
	>30	187	45.1
Residence	Rural	235	56.6
	Urban	180	43.4
Marital status	Married	328	79.0
	Single	51	12.3
	Divorced	36	8.7
Educational status	Unable to read and write	133	32.0
	Read and write	119	28.7
	Primary school	31	7.5
	Secondary school	37	8.9
	College and above	95	22.9
Occupation status	Housewife	218	52.5
	Government employee	73	17.6
	Merchant	36	8.7
	Private employee	88	21.2
Religion	Orthodox	338	81.4
	Muslim	77	18.6

#### Obstetric characteristics of participants

Nearly 85% of study participants had planned pregnancy, and about 35% of study participants had antenatal care (ANC) visits of one to three. Nearly two-thirds of study participants were multigravida, 55.7% were multiparous and nearly 60% of study participants delivered with spontaneous vaginal delivery (Table 5).

**Table 5:** Obstetrics characteristics of study participants of the BEmONC services at Beyeda District, Northwest Ethiopia, 2022 (*n* = 415).

Variables	Category	Frequency	Percent
Planned pregnancy	Yes	351	84.6
	No	64	15.4
Frequency of visits of ANC follow-up	Have no	144	34.7
	One to three	192	46.3
	Four and above	79	19.0
Gravidity	Primigravida	90	21.7
	Multigravida	270	65.1
	Grand multigravida	55	13.3
Parity	Nulliparous	24	5.8
	Primparous	130	31.3
	Multiparous	231	55.7
	Grand multiparous	30	7.2
Mode of delivery	Spontaneous vaginal delivery	358	86.26
	Cesarean section delivery	50	12.5
	Instrumental delivery	7	1.69

### Quality of delivery of BEmNOC services

This study revealed that the implementation fidelity status of quality of delivery was 72.1%. This study also showed that the proportion of clients who perceived that healthcare providers called by name while providing services was 80%. Whereas 73% of clients felt that healthcare providers provided accurate information about BEmONC during service delivery. Furthermore, 59% of clients felt that they were treated with respectful maternity care (RMC) by healthcare providers during the delivery of services (Table 6). The key informant interviews also supported this finding.

**Table 6:** Judgment analysis matrix of quality of delivery dimension indicators for evaluating the implementation fidelity of the BEmONC service at Beyeda District, Northwest Ethiopia, 2022(*n* = 415).

Indicators	E	O	W	S	A (%)	JP
Proportion clients who reported that health care provider called by name while providing services	415	332	4	3.20	80.0	High
The proportion of clients who perceived that healthcare providers provided the right information about BEmONC during service delivery	415	303	5	3.65	73.0	Medium
The proportion of clients who perceived that they received respect from the health care providers during service delivery	415	328	5	2.95	59.0	Low
The proportion of clients who perceived that healthcare providers provided adequate information about the service	415	330	5	3.98	79.6	Medium
The proportion of clients who were satisfied with healthcare providers’ interaction	415	335	5	4.04	80.8	High
The proportion of clients who perceived that healthcare providers answered their questions	415	297	5	3.1	61.6	medium
Overall fidelity of quality of delivery			29	22.4	72.1	Medium

E, expected; O, observed; W, weight; S, score [(observed × weight)/expected]; A, achievement in percentage [(S/W) × 100]; JP, judgment parameter.

*“Our health center's midwives are certainly disciplined and show respect for every client. However, when the work becomes overwhelming, showing respect may become difficult” (Health center head in Beyeda district).*

### Factors associated with quality of delivery

In this study, age, occupational status, and frequency of ANC visits were significantly associated with the quality of delivery of BEmONC services. Those clients whose age is greater than 30 years were 2.81 times judged positively the quality of delivery of BEmONC services provided by health care providers as compared to those clients less than 20 years old (AOR = 2.81, 95% CI: 1.09–7.26). Government-employed clients were 3.65 times positively judged the quality of delivery of the services as compared to housewives (AOR = 3.65, 95% CI: 1.95–6.84). Moreover, mothers who had four and above ANC visits were three times judged positively the quality of delivery of BEmONC services as compared to those have no ANC visit (AOR = 3.34, 95% CI: 1.04–10.69) (Table 7).

**Table 7:** Bivariable and multivariable logistic regression analysis to assess factors associated with quality of delivery of BEMONC services at Beyeda District, Northwest Ethiopia, 2022 (*n* = 415).

Variables	Clients’ judgment status		COR (95% CI)	AOR (95% CI)
	Negative *n* (%)	Positive *n* (%)		
Age in years				
<20	8 (5.4)	25 (9.4)	1	
20–24	20 (13.4)	46 (17.3)	1.34 (0.52–3.56)	1.30 (0.46–3.69)
25–30	47 (31.5)	82 (30.8)	1.79 (0.75–4.29)	2.00 (0.75–5.30)
>30	74 (49.7)	113 (42.5)	2.05 (0.88–4.78)	2.81 (1.09–7.26)*
Educational status				
Unable to read and write	95 (35.7)	36 (24.2)	1	1
Read and Write	52 (34.9)	66 (24.8)	2.08 (1.23–3.53)	0.85 (0.43–1.69)
Primary School	13 (8.7)	18 (6.8)	1.91 (0.85–4.28)	0.79 (0.30–2.06)
Secondary school	10 (6.7)	29 (10.9)	0.91 (0.40–2.06)	0.45 (0.15–1.34)
Collage and above	38 (25.5)	58 (21.8)	1.73 (0.99–3.03)	1.24 (0.48–3.19)
Occupational status				
Housewife	50 (33.6)	146 (54.9)	1	1
Government employee	52 (34.9)	41 (15.4)	3.70 (2.20–6.23)	3.65 (1.95–6.84)*
Merchant	15 (10.1)	21 (7.9)	2.09 (1.00–4.36)	3.16 (0.22–8.18)
Private employee	32 (21.5)	58 (21.8)	1.61 (0.94–2.76)	0.85 (0.33–2.20)
Residence				
Rural	76 (51.0)	159 (59.8)	1	1
Urban	73 (49.0)	170 (40.2)	1.43 (0.95–2.14)	1.00 (0.58–1.74)
Religion				
Orthodox	128 (85.9)	210 (78.9)	1.63 (0.94–2.81)	1.90 (0.99–3.66)
Muslim	21 (14.1)	56 (21.1)	1	
ANC follow-up				
Yes	109 (73.2)	162 (60.9)	1.75 (1.13–2.71)	0.82 (0.25–2.75)
No	40 (26.8)	104 (39.1)	1	1
Frequency of visits				
Have no	37 (24.8)	107 (40.2)	1	1
One to three	74 (49.7)	116 (43.6)	1.85 (1.15,2.96)	2.24 (0.70–7.20)
Four and above	38 (25.5)	43 (16.2)	2.56 (1.44,4.54)	3.34 (1.04–10.69)*

* Statistically significant at *p*-value <0.05, COR, crude odds ratio; AOR, adjusted odds ratio.

## Discussion

The overall implementation level of the BEmONC services was 72.8% which was judged as implemented in medium fidelity. Moreover, adherence, participant responsiveness and quality of delivery of the BEmONC services were found to be implemented in medium fidelity. This finding is inconsistence with the requirements of obstetric triage and emergency care protocol guideline which recommends the fidelity of BEmONC services in each health facility should be implemented fully (35).

This evaluation revealed that partograph was recorded based on obstetrics management protocol for 81% of the study participants. This finding was higher than the study conducted in Tanzania where partograph was documented for 44.4% of deliveries (6). These differences may be due to differences in the adherence of healthcare workers towards utilization of the recommended obstetrics management protocol guidelines.

In this fidelity evaluation, uterotonic drugs, parenteral anticonvulsants and parenteral antibiotics were administered based on the algorism for 58.3%, 69.3% and 81.3% of participants respectively.

This finding was higher than the study conducted in Philippines in which uterotonic drugs, parenteral anticonvulsants and parenteral antibiotics were administered based on the algorism for 52.24%, 32.24% and 39.19% participants respectively (36). This inconsistency may be due to differences in the availability of these essential drugs in health facilities.

Moreover, this evaluation showed that nearly three-fourths (74%), 78.45 and 81.6% of study participants were performed assisted vaginal delivery, removal of retained products and neonatal resuscitation respectively based on the protocol. This finding was higher than the study conducted in Lusaka, Zambia where 31%, 35% and 46% of study participants were performed assisted vaginal delivery, removal of retained products and neonatal resuscitation respectively according to the recommended protocol (37). These differences might be due to differences in the availability of sufficient obstetric guidelines in health facilities.

This evaluation also showed that 56.4% of study participants were interested towards the service delivery of neonatal resuscitation. In this study, it is observed that nearly three-fourths (73%) of study participants perceived that healthcare providers delivered the right information about BEmONC during the service delivery and 59% of study participants perceived that they received respect from the healthcare providers during service delivery. This finding was lower than the study conducted in Tigray, Ethiopia which reported that 88.2% of study participants perceived that healthcare providers delivered the right information about BEmONC during service delivery and 96.2% of study participants perceived that they received respect from the healthcare providers during service delivery (26).

This evaluation revealed that participants whose age is greater than 30 years were 2.81 times judged positively the quality of delivery of BEmONC services provided by health care providers as compared to those clients less than 20 years old (AOR = 2.81, 95% CI: 1.09–7.26). This might be because, as age increases, their perspective about the importance of the services also increases. As a result, they might ask about the services and know what is being done.

Government-employed clients were 3.65 times judged positively the quality of delivery of the services as compared to housewives (AOR = 3.65, 95% CI: 1.95–6.84). This might be because government employees spend most of their time at the workplace and might receive more information from their colleagues about the services. In addition, government employees might be able to get more information regarding the services either through training or other workshops.

Moreover, mothers who had four and above ANC visits were three times judged positively the quality of delivery of BEmONC services as compared to those have no ANC visit (AOR = 3.34, 95% CI: 1.04–10.69). This might be due to repeated ANC visits and frequent contact with HCPs might contribute mothers to having a positive attitude towards the service delivery.

### Strengths and limitations of the evaluation

The strength of the evaluation is that the evaluation employed a mixed-method study design which has integrated both qualitative and quantitative research approaches. A mixed-methods research approach provides a more holistic view, making the findings more relevant and actionable for practitioners, policymakers, and stakeholders. The limitation was that since the study was institutional based, there might be a social desirability bias which might result in relatively high levels of positive response towards the services. To mitigate this issue, data collectors were provided with sufficient training on appropriate interview techniques, and interviewing clients in a separate room were taken as part of the strategy.

## Conclusions

Overall the implementation fidelity of the BEmONC services in this study was judged as implemented in medium fidelity as per the preset judgment parameter. Moreover, the adherence, participant responsiveness and quality of delivery dimensions were found to be implemented in medium fidelity. Age greater than 30 years, being government employed and ANC visits four and above were variables significantly associated with quality of delivery.

Therefore, public health sectors at all levels should strive to enhance the implementation fidelity of BEmONC services. Moreover, healthcare providers should adhere to the BEmONC implementation protocol guideline during service delivery. Healthcare providers also better deliver obstetric and neonatal care services compassionately and respectfully.

## Data Availability

The raw data supporting the conclusions of this article will be made available by the authors, without undue reservation.
